# Therapeutic potential of stem cells from human exfoliated deciduous teeth infusion into patients with type 2 diabetes depends on basal lipid levels and islet function

**DOI:** 10.1002/sctm.20-0303

**Published:** 2021-03-04

**Authors:** Wenwen Li, Xuan Jiao, Jingyun Song, Bingdong Sui, Zhili Guo, Yingji Zhao, Jun Li, Songtao Shi, Qin Huang

**Affiliations:** ^1^ Department of Endocrinology Changhai Hospital, Second Military Medical University Shanghai People's Republic of China; ^2^ South China Center of Craniofacial Stem Cell Research Guanghua School and Hospital of Stomatology, Sun Yat‐sen University Guangzhou People's Republic of China; ^3^ Research and Development Center for Tissue Engineering School of Stomatology, Air Force Medical University People's Republic of China; ^4^ Department of Dermatology Changhai Hospital, Second Military Medical University Shanghai People's Republic of China; ^5^ Easter Greenland Hospital People's Republic of China

**Keywords:** diabetes, glucose metabolism, islet function, mesenchymal stem cells, stem cells from human exfoliated deciduous teeth

## Abstract

Mesenchymal stem cells (MSCs) hold great potential in treating patients with diabetes, but the therapeutic effects are not always achieved. Particularly, the clinical factors regulating MSC therapy in this setting are largely unknown. In this study, 24 patients with type 2 diabetes mellitus (T2DM) treated with insulin were selected to receive three intravenous infusions of stem cells from human exfoliated deciduous teeth (SHED) over the course of 6 weeks and were followed up for 12 months. We observed a significant reduction of glycosylated serum albumin level (*P* < .05) and glycosylated hemoglobin level (*P* < .05) after SHED transplantation. The total effective rate was 86.36% and 68.18%, respectively, at the end of treatment and follow‐up periods. Three patients ceased insulin injections after SHED transplantation. A steamed bread meal test showed that the serum levels of postprandial C‐peptide at 2 hours were significantly higher than those at the baseline (*P* < .05). Further analysis showed that patients with a high level of blood cholesterol and a low baseline level of C‐peptide had poor response to SHED transplantation. Some patients experienced a transient fever (11.11%), fatigue (4.17%), or rash (1.39%) after SHED transplantation, which were easily resolved. In summary, SHED infusion is a safe and effective therapy to improve glucose metabolism and islet function in patients with T2DM. Blood lipid levels and baseline islet function may serve as key factors contributing to the therapeutic outcome of MSC transplantation in patients with T2DM.

## Graphical abstract

Stem cells from human exfoliated deciduous teeth (SHED) infusion is a safe and effective therapy to improve glucose metabolism and islet function in type 2 diabetes mellitus (T2DM) patients. Blood lipid levels and baseline islet function may serve as a key factors contributing to the therapeutic outcome of mesenchymal stem cells transplantation in T2DM patients.

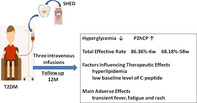

**Lessons learned**

- Infusion of stem cell from human exfoliated deciduous teeth is a safe and effective therapy in patients with type 2 diabetes mellitus.
- The control of blood glucose and blood lipid before infusion is helpful to improve the efficacy of stem cell infusion.
- Patients with residual islet β cell function benefit more from stem cell infusion.

**Significance statement**

Mesenchymal stem cells (MSCs) hold great potential in treating diabetic patients, but the therapeutic effects are not always achieved. Particularly, the clinical factors regulating MSC therapy in this setting are largely unknown. This study confirmed that stem cells from human exfoliated deciduous teeth transplantation are a simple, safe, and effective therapy for diabetic patients. Blood lipid levels and baseline islet function may serve as a key factor contributing to the therapeutic outcome of MSC transplantation in these patients.

## 1 INTRODUCTION

Diabetes is an increasingly serious global epidemic disease.^1^, ^2^ According to the International Diabetes Federation, the global number of patients with diabetes over 20 years old reached 424.9 million in 2017, and the total number of patients is expected to reach 628.6 million by 2045.^1^ Type 2 diabetes mellitus (T2DM) accounts for more than 90% of all cases of diabetes mellitus.^3^ Although the pathogenesis has not been fully elucidated, the dysfunction of β cells plays a key role in its occurrence and development.^4^, ^5^, ^6^ The existing hypoglycemic drugs, including insulin, are mainly used to control hyperglycemia and delay the development of complications but fail to effectively reverse the progressive reduction and failure of islet β cells.^7^, ^8^ Promoting islet β‐cell regeneration and restoring endogenous insulin secretion may present an ideal approach to curing diabetes.^9^, ^10^

Previous studies showed that allogeneic pancreas or islet transplantation can effectively improve islet function in patients with diabetes. Nevertheless, this approach has not been widely used because of the limited donor resources and the possibility of immune rejection, as well as the toxicity of immunosuppressive drugs required to maintain the transplants.^11^, ^12^, ^13^ For the application of stem cell regeneration strategies, the use of embryonic stem cells and induced pluripotent stem cells poses ethical problems and has the risk of tumorigenesis, which also limits their clinical application.^14^, ^15^, ^16^, ^17^ Against this bottleneck issue, mesenchymal stem cells (MSCs) stand out for their great clinical potential, as they may be able to restore the function of islet β cells and have many advantages, such as being easily obtained from accessible sources, limited trauma associated with harvest, and low immunogenicity.^18^, ^19^ Therefore, MSC therapy has attracted great attention in the field of diabetic research.

MSCs have the capacity of self‐renewal, multidirectional differentiation, and secretion of cytokines such as tumor necrosis factor‐induced protein 6,^20^ interleukin‐10,^21^ and nitric oxide.^22^ It has been suggested that MSCs from different sources can differentiate into insulin‐producing cells (IPCs),^23^, ^24^, ^25^ which secrete insulin in a glucose‐dependent manner. In addition, MSCs can migrate to the damaged islets in diabetic mice and reshape the microenvironment to make it suitable for the proliferation and functional recovery of endogenous islet cells, thus enabling repair of the damaged islet function.^26^, ^27^

Dental pulp MSCs derived from adult teeth (dental pulp stem cells [DPSCs]) or deciduous teeth (stem cells from human exfoliated deciduous teeth [SHED]) have the ability to form cell colonies and show strong capacity of proliferation.^28^ It was reported that DPSCs and SHED in culture can be induced into islet cell clusters (ICCs) with expression of pancreatic markers and positivity for dithizone staining, along with secretion of insulin and C‐peptide in a glucose‐dependent manner.^29^, ^30^ Transplantation of SHED‐derived ICCs can rescue blood glucose levels in diabetic mice.^30^ In addition, previous studies showed that SHED are effective for treating type 2 diabetic rats with mechanisms related to improvement of islet β cells and the liver metabolism.^31^, ^32^ Although SHED and DPSCs have many advantages in clinical use, such as their abundant, easy access, low rate of side effects, and lack of ethical disputes, there is no research establishing clinical efficacy of SHED or DPSCs to treat diabetes. In this study, we assess the safety and effectiveness of allogenic SHED transplantation in patients with T2DM and evaluate potential factors regulating therapeutic efficacy of SHED.

## 2 MATERIALS AND METHODS

### 2.1 Subjects

Twenty‐four patients with T2DM were enrolled in the Endocrinology Department of Shanghai Changhai Hospital from May to August in 2018. Inclusion criteria were as follows: (a) age was between 45 and 65 years, and body mass index was between 20 and 30 kg/m^2^. The course of T2DM was more than 5 years. Patients were able to understand the purpose of clinical trial, voluntarily participated, and signed the informed consent. (b) After insulin treatment with or without oral hypoglycemic drugs, the range of fasting blood glucose (FBG) was 7.5 to 12.0 mmol/L, and the range of glycosylated hemoglobin (HbA1c) was 7.0% to 10.0%. (c) Duration of insulin injection was more than 1 year, the frequency of subcutaneous insulin injections was more than twice a day in the past 3 months, and the daily dose of insulin use was ≥0.4 IU/(kg/d). (d) Oral hypoglycemic drug use was limited to metformin, α‐glucosidase inhibitors, or insulin secretagogues for more than 3 months. Exclusion criteria were as follows: (a) T2DM, gestational diabetes mellitus, or any other special types of diabetes mellitus. (b) Acute complications such as diabetic ketoacidosis or nonketotic hyperosmolarity syndrome occurred within 1 month before screening. (c) Patients received other stem cell therapy before screening or had participated in or were participating in another clinical research programs within the past 3 months. (d) Poor blood pressure control and/or blood pressure ≥160/100 mmHg at the time of screening. (e) Use of thiazolidinediones, dipeptidyl peptidase‐IV inhibitors or glucagon‐like peptide‐1 analogs within 3 months before screening. (f) Insulin was used for less than 1 year or subcutaneously injected once a day in the past 3 months before screening. (g) Pancreatic diseases, including a history of acute or chronic pancreatitis or pancreatic tumors. (h) Other malignant tumors or suspected tumor tendency, or patients who were in the active stage of various infectious diseases (including the active stage of hepatitis B or C) or who were HIV positive. (i) Other serious systemic diseases (such as diseases of the cardiovascular system, the respiratory system, the digestive system, the nervous system, the endocrine system, the immune system, and the blood system, etc.). (j) Abnormal liver or kidney function, such as serum total bilirubin exceeding 1.5 times the upper limit of normal value, alanine aminotransferase (ALT) or aspartate aminotransferase (AST) exceeding 2.5 times of the up limit of normal value, or serum creatine (SCr) exceeding 1.2 times the upper limit of normal value. (k) Current use of systemic hormones (such as glucocorticoids), immunosuppressants, or cytotoxic drugs. (l) Pregnant or lactating women, drug users or patients who had a history of adverse drug abuse and alcohol dependence within the past 5 years, and patients who had contraindications or allergies in the treatment of this study. This study was conducted in strict accordance with the relevant laws and regulations such as the Administrative Measures for Stem Cell Clinical Research (trial) issued by the National Health and Family Planning Commission and the State Food and Drug Administration. This study was approved by the ethics committee of Shanghai Changhai Hospital (ethics approval number: CHEC2017‐159; clinical trial registration number: NCT03658655), and all the enrolled patients signed the informed consent forms. Baseline parameters of the patients were recorded when patients were enrolled in the study.

### 2.2 Transplantation and follow‐up

This study was divided into a screening period, a treatment period, and a follow‐up period. The screening period lasted 1 week, and the patients who met the criteria were enrolled within 1 week after screening. The patients received three intravenous infusions of SHED during the 42‐day treatment period. The first infusion was scheduled at the time of enrollment, and the secondary and third infusions were scheduled 1 and 4 weeks after the first infusion, respectively. SHED were provided by CAR‐T (Shanghai) Biotechnology Co., Ltd., and the preparation of stem cells complied with the relevant provisions of the guidelines for quality supervision and preclinical research of stem cell preparations. SHED used in this study were donated with informed consent and were collected from the naturally exfoliated teeth without invasive procedures. The donors of SHED had been tested for biosafety (Table S1). Characterization of SHED was performed according to our previous studies by morphology, surface markers, and multidifferentiation function.^33^ In addition, SHED used in this study had also passed the tumorigenic tests before the infusion. The dosage of each stem cell infusion was calculated as 0.1 U/kg body weight, in which each unit contains 1 × 10^7^ stem cells. The dosage of SHED infusion was selected based on our previous studies using MSCs to treat patients with autoimmune disorders.^34^, ^35^, ^36^ The follow‐up periods included examinations at 1, 2, 3, 6, 9, and 12 months after the third SHED infusion. During the study period, the insulin dosage was adjusted according to the changes in blood glucose levels, whereas the types and doses of oral hypoglycemic drugs were not adjusted if the patients had no side effects or had ceased insulin injections.

### 2.3 Research methods

On the day of enrollment, we provided self‐management training to all enrolled patients, including information on the importance of diet and exercise to diabetes and common symptoms of hypoglycemia. A unified blood glucose monitor (model GE333D, Huaguang Biotechnology, Pingtan, China) was issued to the patients. During the follow‐up period, the self‐management awareness and ability of patients were checked and strengthened. Routine blood and urine panels, blood total cholesterol (TC), triglycerides (TG), high‐density lipoprotein cholesterol (HDL‐C), low‐density lipoprotein cholesterol (LDL‐C), AST, ALT, SCr, estimated glomerular filtration rate (eGFR), HbA1c and/or glycosylated serum albumin (GSP), steamed bread meal test, and insulin release test were conducted at each follow‐up point. A daily life diary was distributed. Patients were required to record their diet and exercise, capillary blood glucose (CBG) monitoring value, daily insulin dose and oral medicine use, hypoglycemia, and other adverse reactions, if any, in detail. The diary was then collected at each follow‐up visit. Commonly used CBG monitoring time points included fasting, preprandial, 2 hours after each of the three daily meals, bedtime, and any time of discomfort. In this study, the daily dose of insulin was taken as the main evaluation index.^37^, ^38^ When the daily dose of insulin was reduced by ≥50% compared with the baseline, the intervention was assessed as remarkably effective; when the daily dose of insulin was reduced by ≥20% but <50% compared with the baseline, it was assessed as moderately effective; when the daily dose of insulin was reduced by <20% compared with the baseline, it was assessed as ineffective. Changes in HbA1c, FBG, and continuous blood glucose monitoring results were used as secondary efficacy evaluation indexes. The insulin resistance index (HOMA‐IR) was calculated by the equation FBG (mmol/L) × fasting insulin (mmol/L) /22.5.

### 2.4 Statistical analysis

SPSS 23.0 statistical software was used. The data are expressed as means ± SD, and the counting data are expressed as number of cases and percentage. Analysis of variance and Student‐Newman‐Keuls q (SNK‐q) test were used for comparisons between groups, and the significance level (*α* or *P*) was <.05.

## 3 RESULTS

### 3.1 Metabolic indexes

**FIGURE 1:**
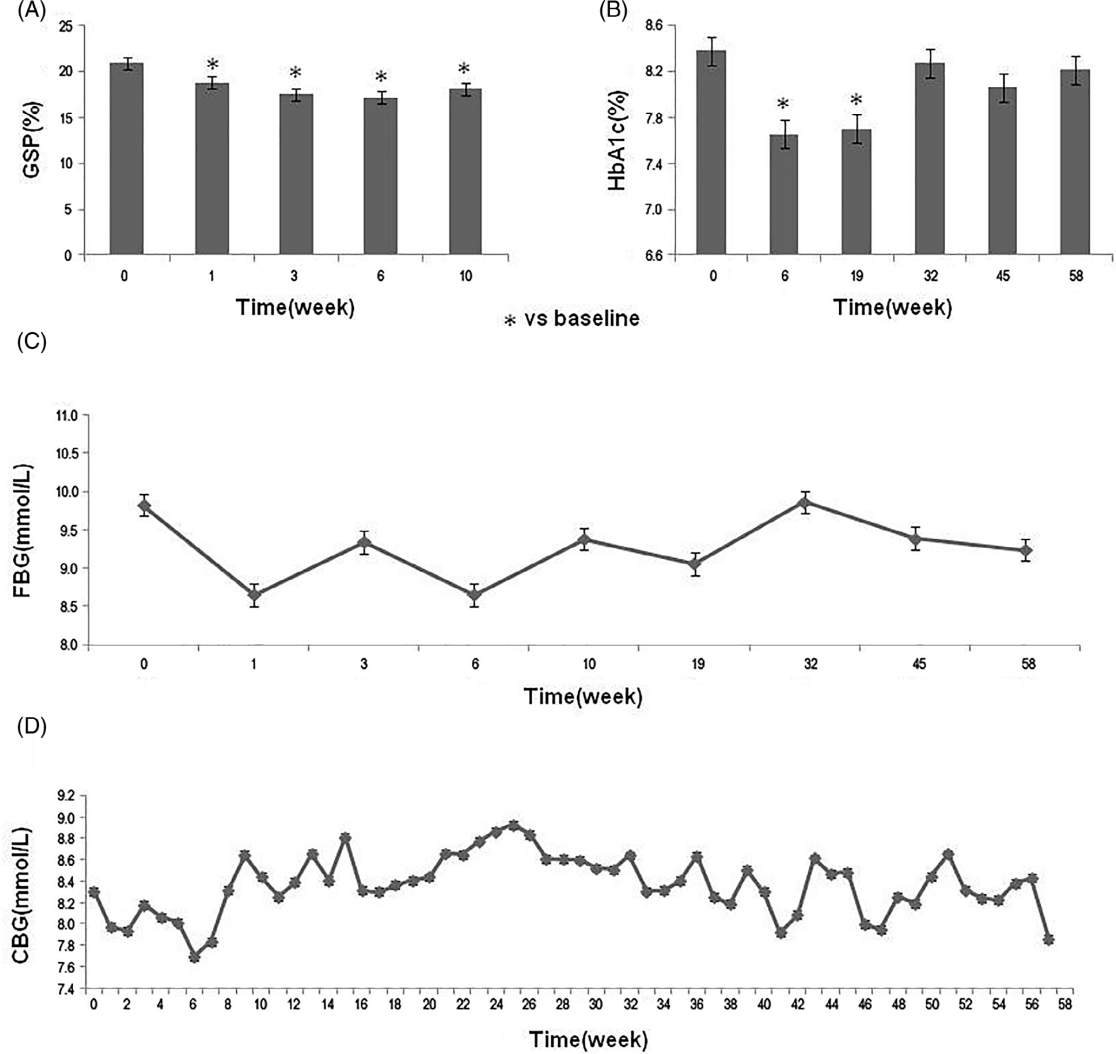
Changes of the glucose metabolism indexes during the study period. A, Patients' GSP levels from baseline to 1 month after the end of treatment period. *P < .05 vs the baseline level. B, Changes of the HbA1c levels during the study period. *P < .05 vs the baseline level. C,D, Patients' FBG levels (C) and self‐monitored fasting CBG values (D) during the study period. CBG, capillary blood glucose; FBG, fasting blood glucose; GSP, glycosylated serum albumin; HbA1c, glycosylated hemoglobin

Among the 24 enrolled patients with T2DM, 2 patients quit after the third follow‐up visit. The patients' baseline information is shown in Table 1. SHED were characterized by assessing typical MSC phenotypes including the morphology, surface markers, and multidifferentiation function (Figure S1). During the treatment period, patients' GSP levels showed a significant reduction compared with the baseline level (*P* < .05). One month after the end of the treatment period, the GSP levels were also significantly lower than the baseline level (*P* < .05) and similar to the values at the end of the treatment period (Figure 1A). The HbA1c levels also reduced significantly when compared with the baseline level at 2 weeks after the end of treatment (*P* < .05). This reduction in HbA1c was maintained until the third follow‐up (12 weeks post the third SHED infusion). After that, the HbA1c level was still lower than the baseline level until the end of the follow‐up period, but there was no statistical difference (Figure 1B). During the treatment period, the FBG level was significantly lower than that of the baseline and then showed an upward trend with no statistical difference compared with the baseline level (Figure 1C). However, patients' self‐monitored fasting CBG values fluctuated slightly during the follow‐up period, and there was no statistical difference (Figure 1D).

**TABLE 1:** General condition of 24 patients at baseline

Index	Minimum	Maximum	Mean
Age (years)	48.00	64.00	55.96 ± 4.81
BMI (kg/m^2^)	20.25	29.80	24.42 ± 2.64
HbA1c (%)	7.20	9.80	8.38 ± 0.74
FBG (mmol/L)	5.80	14.60	9.10 ± 2.68
P2hBG (mmol/L)	13.00	26.60	19.31 ± 4.04
FIN (mIU/L)	1.80	42.90	13.00 ± 10.32
P2hIN (mIU/L)	2.20	143.30	27.61 ± 28.54
FCP (ng/mL)	0.01	2.96	1.44 ± 0.76
P2hCP (ng/mL)	0.01	6.99	3.22 ± 1.58
ALT (U/L)	7.00	66.00	20.79 ± 11.05
AST (U/L)	10.00	30.00	17.88 ± 4.10
TC (mmol/L)	2.81	9.26	5.55 ± 1.46
TG (mmol/L)	0.54	3.80	1.50 ± 0.79
HDL‐C (mmol/L)	0.93	1.89	1.29 ± 0.28
LDL‐C (mmol/L)	1.04	6.95	3.54 ± 1.39
eGFR (mL/min)	46.10	171.80	107.30 ± 27.39
SCr (μmol/L)	37.00	144.00	70.79 ± 19.99
Oral medicine			
α‐glucosidase inhibitor	5/24		
Metformin	9/24		
Metformin + α‐glucosidase inhibitors	2/24		

Abbreviations: ALT, alanine aminotransferase; AST, aspartate aminotransferase; BMI, body mass index; eGFR, estimated glomerular filtration rate; FBG, fasting blood glucose; FCP, fasting C‐peptide; FIN, fasting insulin; HbA1c, glycosylated hemoglobin; HDL‐C, high‐density lipoprotein cholesterol; LDL‐C, low‐density lipoprotein cholesterol; P2hBG, 2‐hour postprandial blood glucose; P2hCP, 2‐hour postprandial C‐peptide; P2hIN, 2‐hour postprandial insulin; SCr, serum creatine; TC, total cholesterol; TG, triglycerides.

**FIGURE 2:**
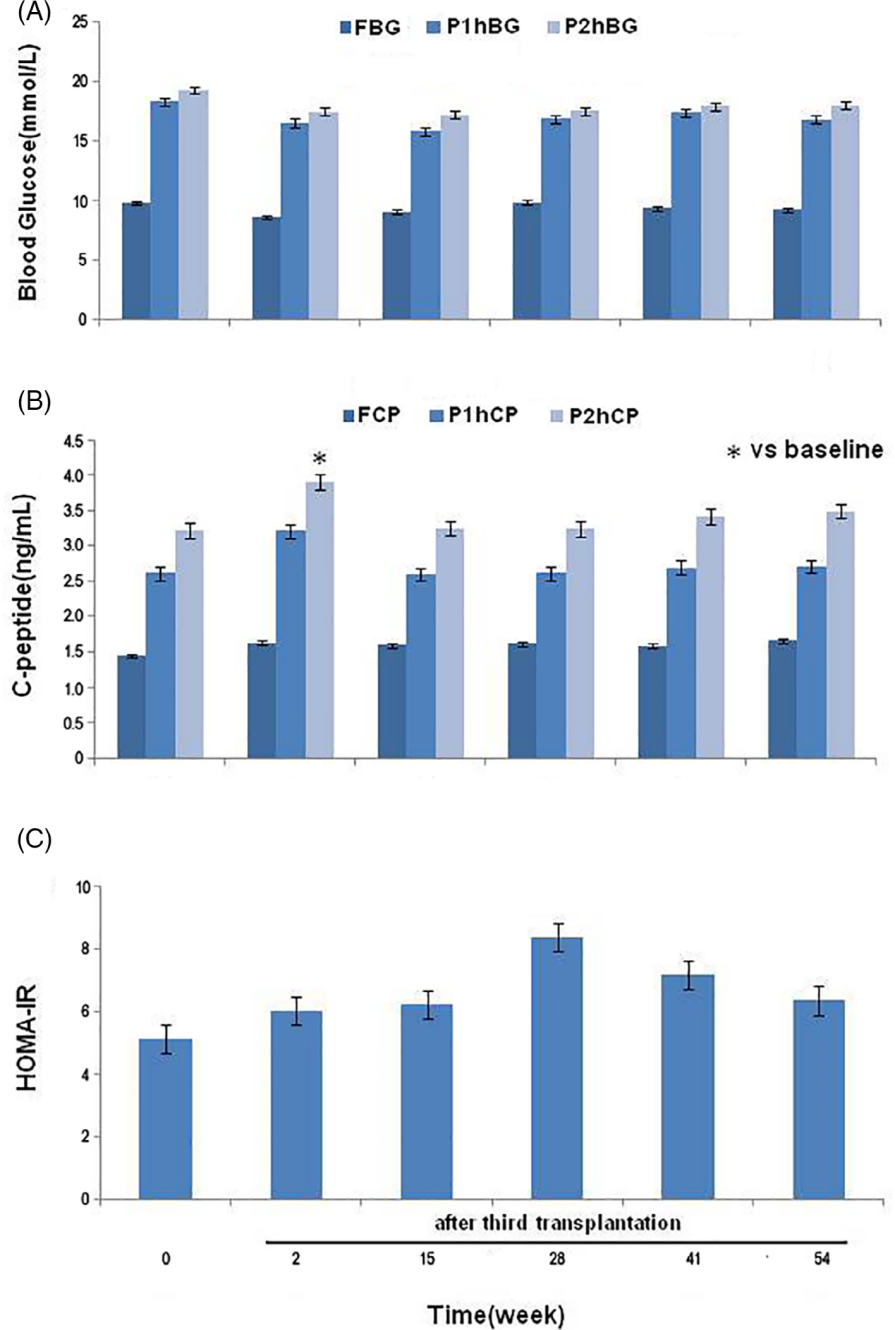
Changes of the glucose metabolism and islet function in the steamed bread meal test. A, Patients' FBG, P1hBG, and P2hBG levels at the baseline, the end of the treatment period and 3, 6, 9, and 12 months after the third infusion of stem cells from human exfoliated deciduous teeth. B, Changes of the FCP, F1hCP, and F2hCP levels during the study period. *P < .05 vs the baseline level. C, Changes of patients' HOMA‐IR during the study period. FBG, fasting blood glucose; FCP, fasting C‐peptide; HOMA‐IR, insulin resistance index; P1hBG, 1‐hour postprandial blood glucose; P1hCP, 1‐hour postprandial C‐peptide; P2hBG, 2‐hour postprandial blood glucose; P2hCP; 2‐hour postprandial C‐peptide

A steamed bread meal test was performed before starting the first SHED infusion, at the end of the treatment period, and 3, 6, 9, and 12 months after the third SHED infusion. At the end of the treatment period and during the follow‐up period, the levels of 1‐ and 2‐hour postprandial blood glucose were lower than the baseline levels and reached the lowest level at 3 months after the end of the treatment period, but the difference was not statistically significant (Figure 2A). SHED transplantation resulted in an elevated level of fasting C‐peptide (FCP), but the difference was not statistically significant (Figure 2B). At the end of the treatment period, the levels of C‐peptide measured at 2 hours postprandially (P2hCP) were significantly higher than those at the baseline (*P* < .05). After that, it showed a downward trend but was still higher than the baseline level at the end of the follow‐up period with no statistical difference (Figure 2B). HOMA‐IR was higher than the baseline level at the end of the treatment period and during the follow‐up period, but the difference was not statistically significant (Figure 2C).

**FIGURE 3:**
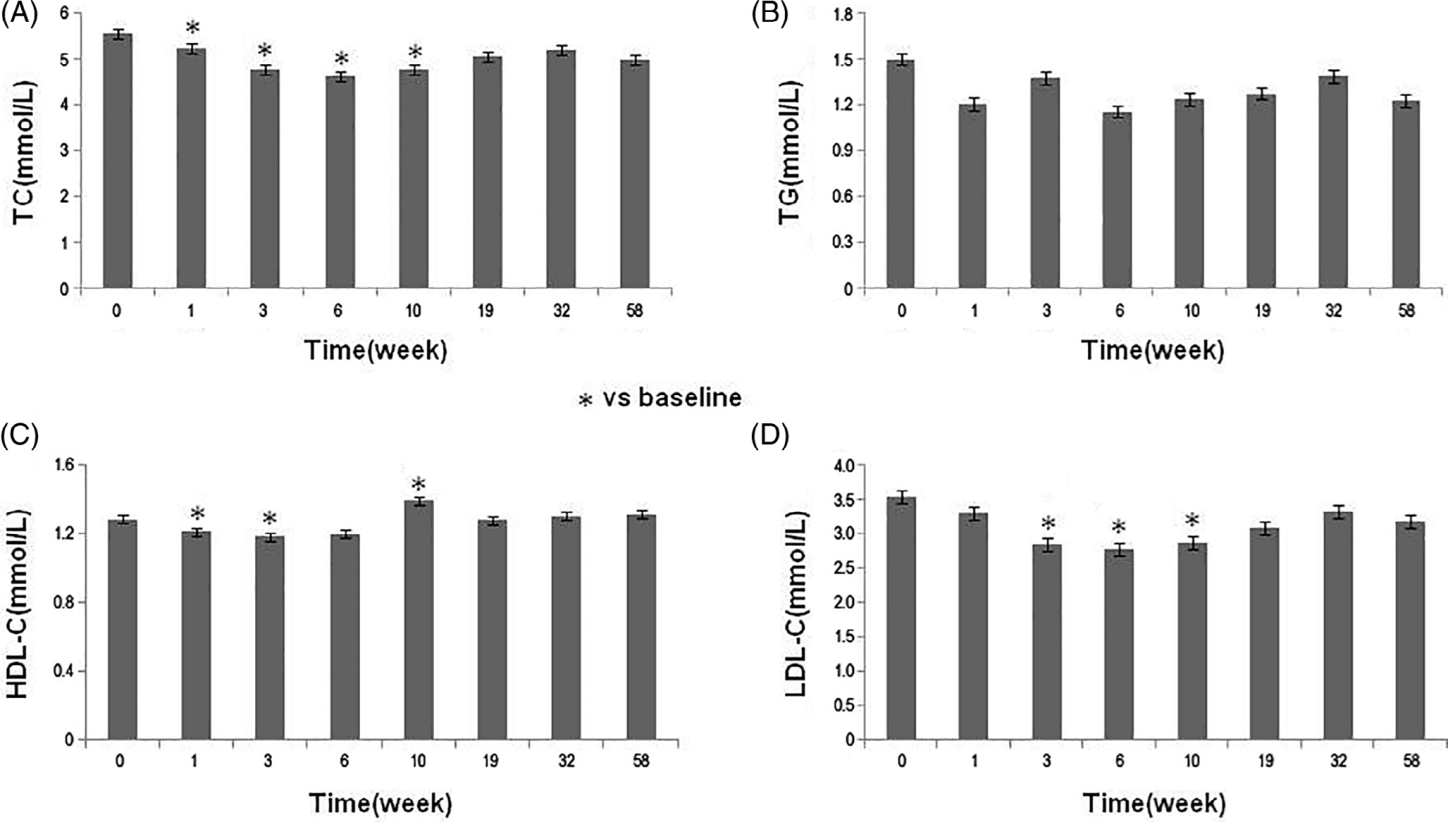
Changes of blood lipids in patients during the study period. Patients' TC, A, TG, B, HDL‐C, C, and LDL‐C levels, D, during the study period. *P < .05 vs the baseline level. HDL‐C, high‐density lipoprotein cholesterol; LDL‐C, low‐density lipoprotein cholesterol; TC, total cholesterol; TG, triglycerides

The levels of TC and LDL‐C decreased gradually after SHED transplantation, and the difference compared with the baseline levels was statistically significant (*P* < .05) at 2 weeks after the second and third SHED transplantation, respectively. The levels of blood TG showed no significant alterations during the observation (Figure 3). The levels of ALT, AST, eGFR, and SCr were within the normal baseline ranges and showed no significant alterations during the follow‐up period (data not shown).

### 3.2 Therapeutic effects

**FIGURE 4:**
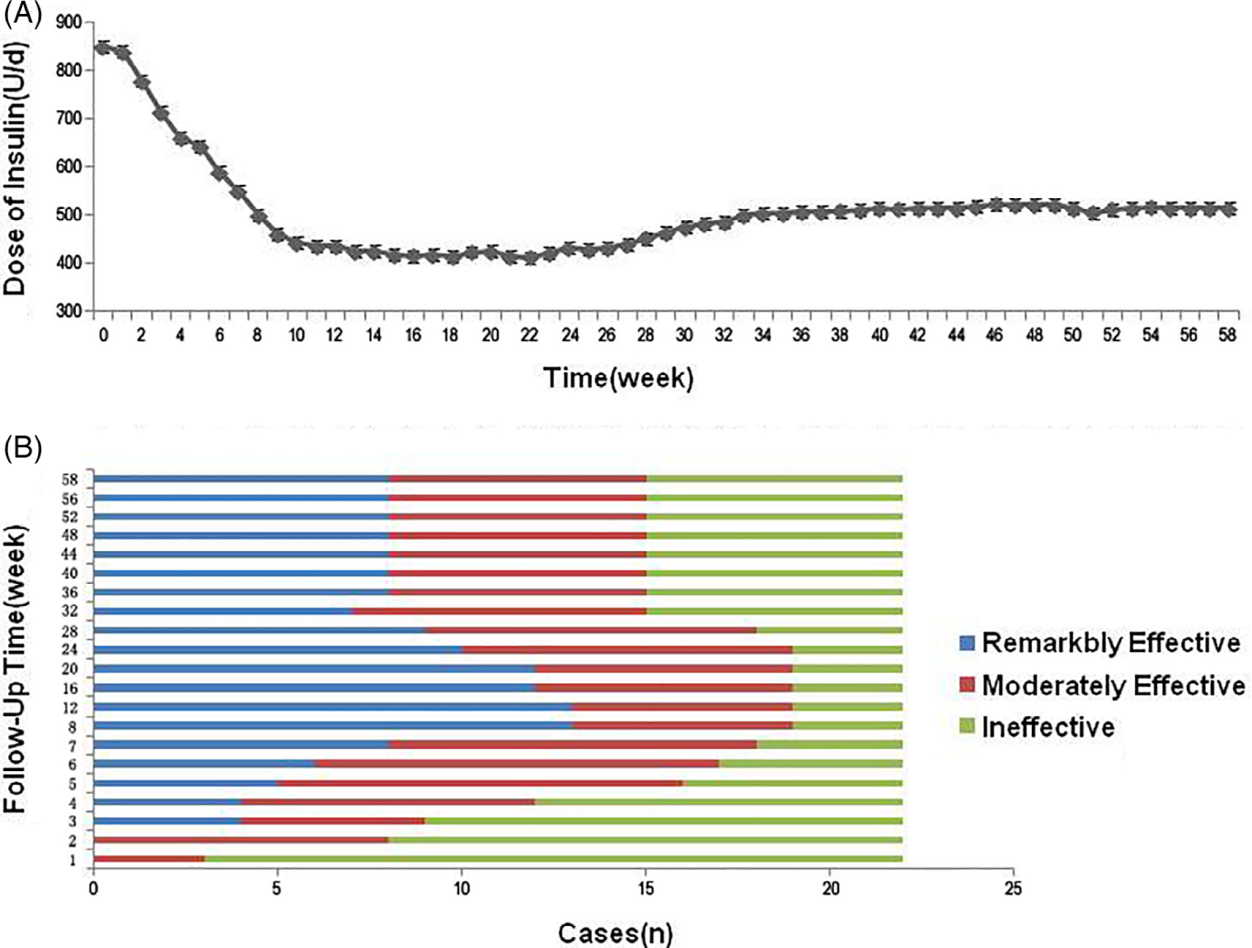
Analysis of the therapeutic effects of stem cells from human exfoliated deciduous teeth (SHED) transplantation therapy. A, Changes of daily insulin dose in 24 patients during the study period. B, Analysis of the efficiency of SHED transplantation therapy in 22 patients

A total of 22 patients completed the study, and their information was used to assess the amount of insulin used by patients on a daily basis. The results showed that the daily dose of insulin reduced significantly over the treatment period, with a decrease of 35.34% at the end of the treatment period compared with the baseline. During the follow‐up period, the daily dose of insulin further reduced. Compared with the baseline level, the daily dose of insulin at 3, 6, and 9 months after the end of the treatment period decreased by 51.18%, 43.39%, and 39.39%, respectively. After that, the dose was relatively stable with no significant reduction (Figure 4A).

The daily insulin dose is the main efficacy evaluation standard of this study. The total effective rate at the end of the treatment period was 86.36% (19/22). Two patients ceased insulin injections during the treatment period. The total effective rate was 81.82% (18/22) at 6 months after the end of the treatment period. Another patient ceased insulin injections during the follow‐up period. By the end of the study, the total effective rate was 68.18% (15/22) with three patients having ceased insulin injections (Figure 4B). In the other four patients, the frequency of insulin injection was also decreased compared with that of baseline.

### 3.3 Correlation analysis

**FIGURE 5:**
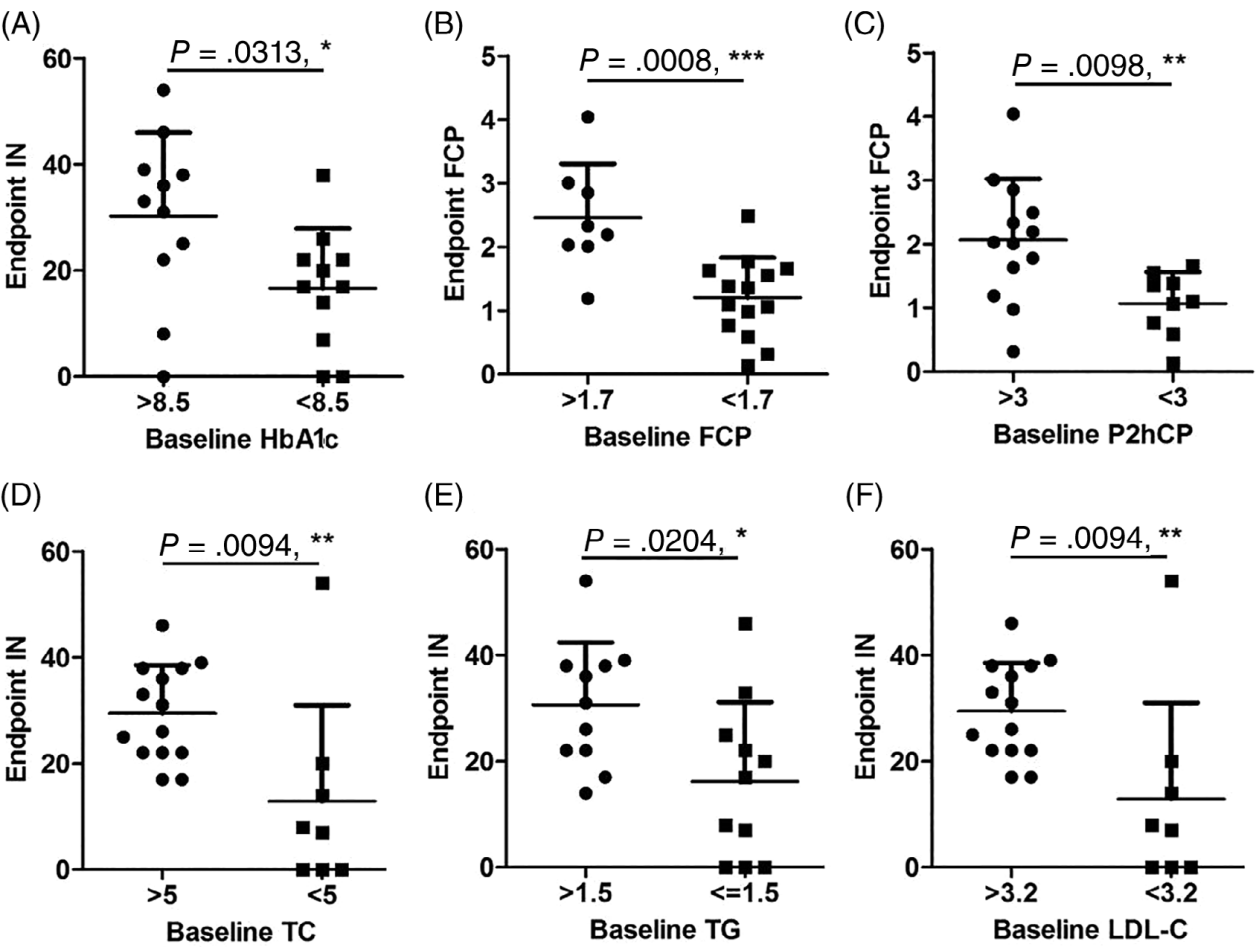
Correlation analysis on the effects of transplantation therapy with stem cells from human exfoliated deciduous teeth. A, HbA1c level before stem cell transplantation affected daily insulin dose at the end of the study. B,C, Baseline FCP (B) and P2hCP (C) levels affected the FCP level at the end of the study. D‐F, Patients' TC (D), TG (E), and LDL‐C (F) levels before treatment affected the daily insulin dose at the end of the study. FCP, fasting C‐peptide; HbA1c, glycosylated hemoglobin; IN, insulin; LDL‐C, low‐density lipoprotein cholesterol; P2hCP, 2‐hour postprandial C‐peptide; TC, total cholesterol; TG, triglycerides

After the threshold of each index was determined by the data distribution map, the initial and endpoint two‐factor correlation analysis were carried out. The results showed that the blood glucose level before stem cell transplantation was correlated with the efficacy; that is to say, enrolled patients with HbA1c <8.5% reduced the daily insulin dose significantly after treatment (Figure 5A). The islet function state of the patients before treatment was closely related to the degree of islet function recovery after treatment, such that patients with FCP >1.7 ng/mL and P2hCP >3 ng/mL showed better islet function recovery after treatment (Figure 5B,C). In addition, patients with TC <5 mmol/L or TG ≤1.5 mmol/L or LDL‐C < 3.2 mmol/L before stem cell therapy showed significant decreases in the daily insulin dose (Figure 5D‐F).

### 3.4 Adverse effects

**FIGURE 6:**
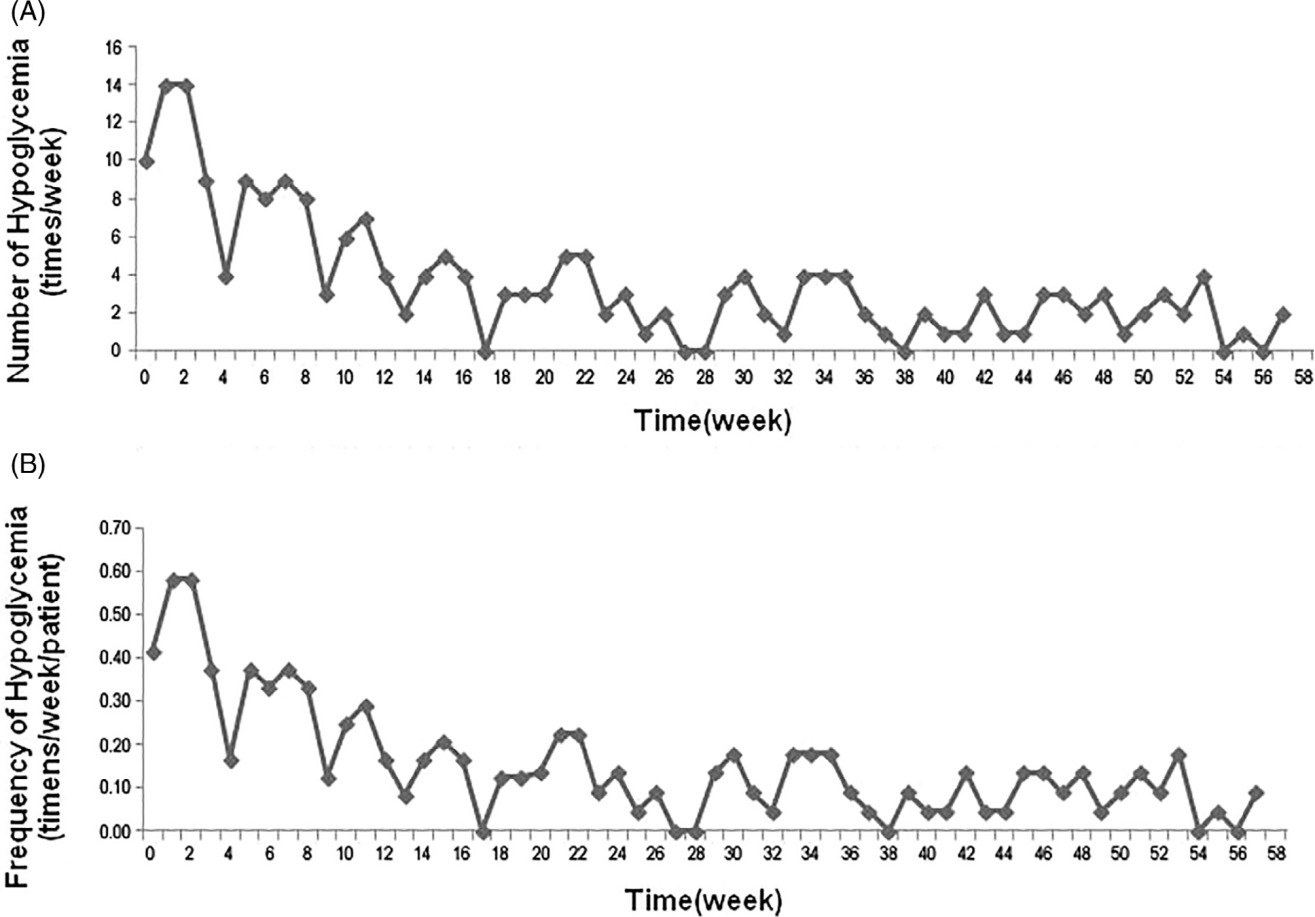
Incidence of hypoglycemia during the study period. A, Hypoglycemia episodes in all patients. B, Frequency of hypoglycemia in each patient

Hypoglycemia was the most common adverse event during the study. The frequency of hypoglycemia in all 24 patients increased during the treatment period and reached a peak of 0.51 times per week per patient. In the follow‐up period, the frequency of hypoglycemia in all 22 patients was significantly reduced. Six months after SHED transplantation, the average frequency of hypoglycemia was about 0.09 times per week per patient, similar to the pretreatment level (Figure 6A,B).

In addition to hypoglycemia, other common adverse reactions during the treatment period were transient fever (11.11%), fatigue (4.17%), and rash (1.39%). All of these adverse reactions occurred within 24 hours after SHED transplantation and were relieved after appropriate standard treatment for the symptoms. During the follow‐up period, one patient was diagnosed with cervical spondylosis, scapulohumeral periarthritis, and fundus hemorrhage. Another two patients suffered from bipedal rupture, with an incidence of 8.33% (2/24). These diseases and their incidence were not correlated with stem cell therapy.

## 4 DISCUSSION

SHED are MSCs that are harvested from the exfoliated deciduous teeth of children, which possess an extremely high capacity of proliferation. In this study, we examine whether SHED can improve glucose metabolism and islet β‐cell function in patients with T2DM. Our results show that SHED transplantation can improve blood glucose and lipid levels in patients with T2DM who have routinely used insulin injection. In addition, SHED transplantation can partially improve islet β‐cell function and significantly reduce the daily insulin dose; some patients ceased insulin injections.

Previous studies showed that stem cell therapy can effectively control the level of blood glucose in patients with T2DM and improve their hyperglycemic status.^38^, ^39^, ^40^, ^41^ Data from multiple centers showed that MSC transplantation significantly reduced HbA1c, but not FBG, levels.^39^ In our study, GSP and HbA1c levels were significantly improved after SHED transplantation. Particularly, HbA1c levels were significantly lower in the treatment period and the first 3 months of the follow‐up period when compared with the baseline level, suggesting that SHED can effectively improve the islet β‐cell function and the level of blood glucose over a relatively long period of time, consistently with previous reports.^38^, ^40^, ^41^ In general, it is difficult to maintain an improved level of HbA1c after MSC treatment, which may be related to multiple factors such as the MSC source, dose, and transplantation frequency, as well as the recipient immune microenvironment. The detailed mechanisms underlying these findings need to be further studied.

There is no standard method for determining the efficacy of stem cell therapy in diabetes. In this study, the daily dose of insulin was used as the main index to evaluate the efficacy of stem cell therapy.^37^, ^38^ The results showed that the daily insulin dose decreased gradually from the beginning of treatment and reached its lowest level at 3 months after the treatment period. At the end of the 1‐year follow‐up period, the daily dose of insulin was still only 60.63% of the baseline level in patients who still used insulin, and three patients had ceased insulin injections altogether. At the end of the treatment period the total effective rate was 86.36%, and it was 68.18% at the 1‐year follow‐up, suggesting that the therapeutic effects of SHED can be maintained for a relatively long period of time. The levels of GSP and HbA1c decreased significantly during the treatment period, indicating potential improvement in the glucose metabolism. Because insulin dose was the primary outcome for evaluating therapeutic effects on T2DM, we showed stable reduction in insulin use after SHED infusion within the follow‐up period. These results implicate that SHED therapy may be a promising approach for T2DM treatment. In addition, it is possible that multiple factors may affect the glucose metabolism, including the lifestyle, emotion, and complications of the patients. Higher frequency of SHED infusion and/or synergistic modulation of recipient lipid microenvironments might be candidates to further improve the glucose metabolism. Considering that the types and doses of oral hypoglycemic drugs were not adjusted in the experimental period, there would have limited effects in terms of influencing the outcome of this study. Nevertheless, these drugs such as metformin may improve SHED function, as documented in vitro,^42^, ^43^ which might exert synergistic effects to alleviate T2DM.^44^, ^45^ Previous studies showed that MSC transplantation can significantly reduce the insulin dose needed by patients with diabetes, or even help them cease insulin injections.^37^, ^38^, ^40^, ^46^ However, these studies used different types of MSCs with different dosages and frequencies of administration and were tested in relatively small numbers of patients. Therefore, a larger sample and a longer period of clinical follow‐up are needed to determine the appropriate dosage and treatment course for stem cell‐based therapy in patients with diabetes.

The mechanisms underlying MSC‐mediated therapy for diabetes have not been elucidated. It has been suggested that MSCs may directly differentiate into IPCs,^23^, ^24^, ^25^ promote islet β‐cell regeneration,^26^, ^27^, ^31^ and improve insulin resistance,^31^, ^47^, ^48^, ^49^ thus playing an important role in improving the glucose metabolism. In addition, regulation of the interplay between immune response and the microenvironment of islet cells may contribute to MSC‐mediated therapy for diabetes.^31^, ^50^, ^51^, ^52^ For the potential mechanisms underlying SHED treatment of T2DM, differentiation of MSCs into pancreas islet after the infusion might be restricted because of limited pancreas homing and rapid apoptosis of the infused cells.^20^, ^53^, ^54^ Nevertheless, infused MSCs are still capable of inducing recovery of β cells based on microenvironmental modulation.^55^, ^56^ SHED‐mediated immunomodulation and islet function restoration have been documented in our previous reports, which potentially contribute to treatment of T2DM.^31^, ^56^ In this study, we also observed the improvement of islet function after SHED transplantation. The results showed that SHED transplantation significantly elevates the level of postprandial C‐peptide while significantly reducing postprandial blood glucose, suggesting that SHED can partially rescue islet function in patients with T2DM. This is consistent with the previous report that MSCs can differentiate into IPCs and secrete insulin in a glucose‐dependent manner.^25^ However, some previous studies showed that MSC transplantation failed to improve islet function in patients with T2DM.^40^, ^46^ The effects of stem cell transplantation on FCP levels have been controversial,^57^ though MSCs appear to improve the FCP levels in patients with T2DM.^37^, ^39^, ^40^, ^41^ Furthermore, infused MSCs can secret a series of paracrine signals such as cytokines and extracellular vesicles, which may regulate hepatic metabolisms to alleviate insulin resistance.^58^, ^59^, ^60^ Nevertheless, in our study, SHED transplantation did not significantly improve insulin resistance, which is consistent with a previous report.^41^ However, previous animal experiments suggested that MSC transplantation can rescue insulin resistance in T2DM rats and mice.^37^, ^38^, ^61^ Our previous animal experiment also showed that the HOMA‐IR of T2DM rats decreased significantly after intravenous infusion of SHED.^31^ The differences in the results of clinical trials and animal experiments with respect to the effects of stem cell therapy on islet function and insulin sensitivity may be due to species‐specific differences in response and differences in the source, dosage, and treatment course of MSCs used. Especially for those who have a long course of disease and have used insulin, it cannot reflect the real insulin resistance. In addition, immunorejection is not a common clinical response of infused allogenic MSCs, because they are hypoimmunogenic and show reliable immunological compatibility with recipients.^18^, ^62^ Infused MSCs can further modulate recipient immune responses to establish an immune tolerance status.^22^, ^63^ Therefore, further research is needed to elucidate the detailed mechanisms.

It is unknown whether MSC transplantation affects blood cholesterol in patients with T2DM. However, MSC transplantation can reduce TC and very‐low‐density lipoprotein cholesterol (VLDL‐C) levels and stabilize atherosclerotic plaques in patients with atherosclerosis.^64^, ^65^ We found that intravenous SHED transplantation can effectively improve the blood lipid metabolism in T2DM rats.^31^ In addition, MSC transplantation may suppress Kupffer cell activity to downregulate VLDL‐C levels.^64^ It is known that Kupffer cells can express mediators that promote hepatocytes to secrete VLDL‐C.^66^ However, the exact mechanisms by which MSCs can improve dyslipidemia have not been elucidated. In this study, we evaluated the peripheral venous blood lipid spectrum and found that the levels of TC and LDL‐C decreased significantly after treatment, suggesting that SHED may have the effect of lowering blood cholesterol levels. This is beneficial to prevent the occurrence and progression of diabetic macrovascular complications and also to achieve the goal of comprehensive management of diabetes. Long‐term blood glucose and islet function improvements are the ultimate goal of T2DM therapy. In this study, although SHED therapy achieved positive clinical effects, its long‐term therapeutic effect is still unknown. We found that the levels of blood lipid affect the efficacy of SHED therapy. Therefore, it is necessary to examine whether reduced blood lipid level improves SHED‐mediated therapeutic effects in T2DM.

Hypoglycemia is a common side effect of MSC‐mediated treatment of diabetes, especially in patients using insulin injection. The frequency of hypoglycemia during the treatment period was significantly higher than that in the follow‐up period in our study, suggesting that the frequency of blood glucose monitoring should be increased during the treatment period and the hypoglycemic treatment plan should be considered carefully. In this study, we strengthened key diabetic knowledge (such as how to identify hypoglycemia and strategies, etc.) and provided individualized education at each follow‐up to strengthen their self‐management ability. Patients were also asked to monitor their fasting and postprandial CBG every day and to increase the frequency of continuous blood glucose monitoring in order to identify asymptomatic hypoglycemia in time to address it. We adjusted the insulin dosage promptly according to the blood glucose levels to reduce the occurrence of hypoglycemia. Other main adverse reactions to SHED transplantation were transient fever, fatigue, and rash, but the incidence of each was low. All adverse reactions occurred within 24 hours after infusion and were relieved upon treatment of the symptoms. We did not observe complications such as headache, nausea and vomiting, which have been reported previously in MSC therapy.^28^ For the fates of systemically infused MSCs, previous studies showed that infused MSCs migrate to different organs with limited capability of homing, and they are mostly trapped in the lung.^20^ Recent studies showed that apoptosis of infused MSCs occurred at 4 hours to 3 days after the infusion^53^, ^54^ and that the apoptotic process plays an important role in MSC‐mediated immune therapies.^54^, ^67^, ^68^ Similarly, no adverse effects on liver or kidney function were observed in this study, indicating that SHED transplantation is a safe therapy.

Because deciduous tooth is the only exfoliated human organ, it contains SHED that offers a unique stem cell resource for clinical application. This is a proof‐of‐concept study with certain degree of therapeutic effects being revealed, and it is necessary to further understand the detailed mechanisms of stem cell‐mediated therapy in diabetes to improve the efficacy. Thus, one of the important findings in this study is to show the blood lipid level influences the results of SHED treatment of T2DM, which will provide a clue for improving SHED therapy in the future. Furthermore, the dosage and frequency of SHED infusion should be adjusted in future studies to safeguard long‐term SHED efficacy in the chronic contexts of diabetes. However, the optimal treatment approach, especially the infusion dose, treatment course, adaptive population, and long‐term efficacy, needs to be confirmed by large‐scale and long‐term clinical and basic research.

## CONFLICT OF INTEREST

The authors declared no potential conflicts of interest.

## AUTHOR CONTRIBUTIONS

W.L., J.S.: provision of study material or patients, collection and/or assembly of data; X.J.: data analysis and interpretation, manuscript writing; B.S.: conception/design, data analysis and interpretation, manuscript writing; Z.G.: collection and/or assembly of data, data analysis, and interpretation; Y.Z.: manuscript writing; J.L.: provision of study material or patients; S.S.: conception/design, final approval of manuscript; Q.H.: conception/design, provision of study material or patients, collection and/or assembly of data, data analysis and interpretation, manuscript writing, final approval of manuscript.

## Supporting information


**Figure S1** Characterization of SHED. A, Morphology of cultured SHED. B, Osteogenic differentiation. C, Adipogenic differentiation. D, Chondrogenic differentiation. E‐L, Flow cytometric analysis of surface markers

**Table S1** Conditions of donors of SHED

## Data Availability

All data generated or analyzed in this study are included in this manuscript.
