# Supplementary figures and images for: Therapeutic potential of stem cells from human exfoliated deciduous teeth infusion into patients with type 2 diabetes depends on basal lipid levels and islet function
Source: Stem Cells Transl Med. 2021 Mar 4;10(7):956–67. doi: 10.1002/sctm.20-0303 (PMC8235136; doi:10.1002/sctm.20-0303)

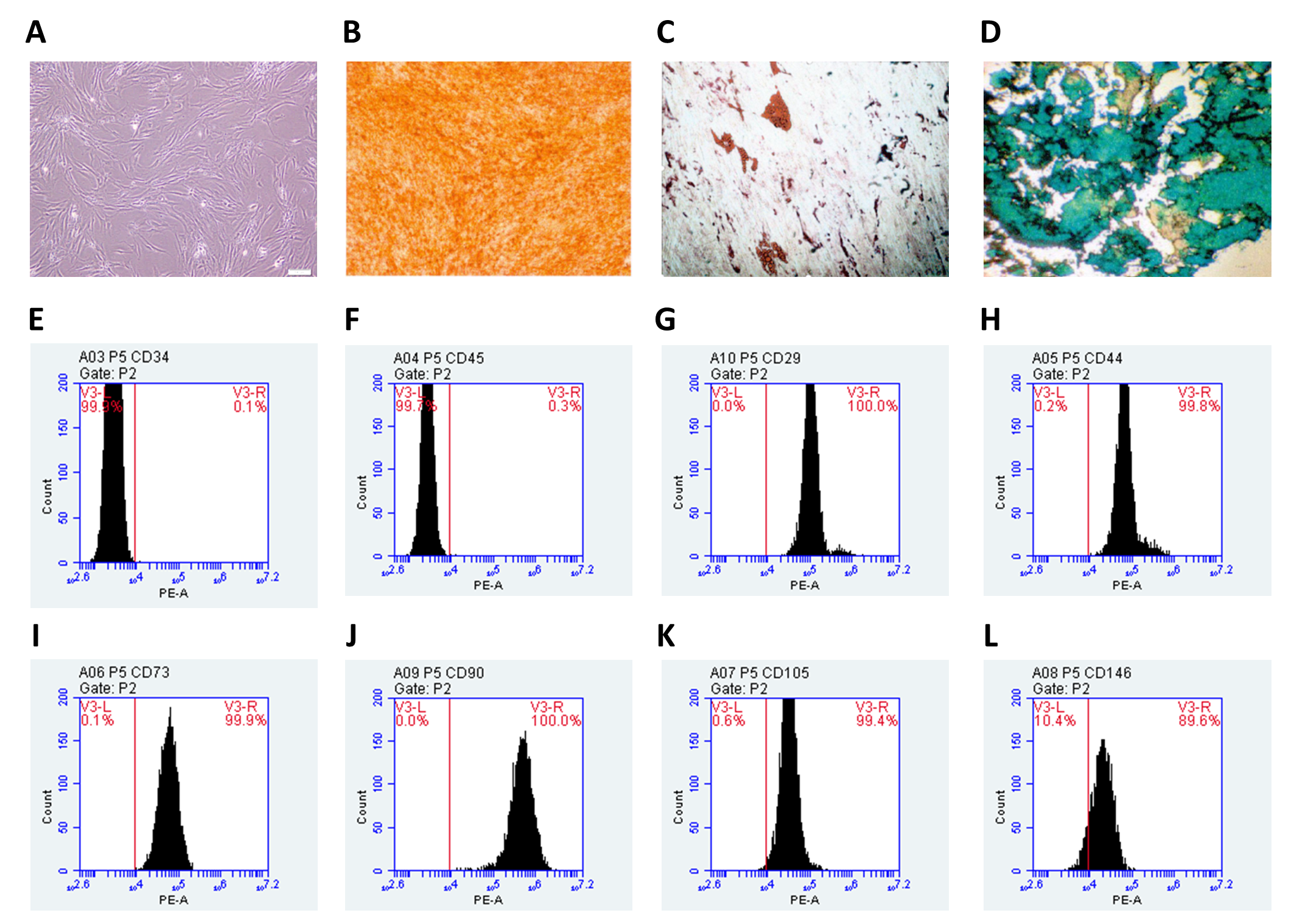

Supplement: Supplementary file 1 — Figure S1 Characterization of SHED. A, Morphology of cultured SHED. B, Osteogenic differentiation. C, Adipogenic differentiation. D, Chondrogenic differentiation. E‐L, Flow cytometric analysis of surface markers [file SCT3-10-956-s001.tif]
