# Supplementary material for: Therapeutic potential of stem cells from human exfoliated deciduous teeth infusion into patients with type 2 diabetes depends on basal lipid levels and islet function
Source: Stem Cells Transl Med. 2021 Mar 4;10(7):956–67. doi: 10.1002/sctm.20-0303 (PMC8235136; doi:10.1002/sctm.20-0303)
Supplement: Supplementary file 2 — Table S1 Conditions of donors of SHED [file SCT3-10-956-s002.docx]

**Supplementary Table**

**Supplementary Table 1 Conditions of donors of SHED.**

| **No.** | **Virus** | **Examination Item** | **Method** | **Criterion** |
| --- | --- | --- | --- | --- |
| 1 | HIV | Human immunodeficiency virus antibody | ELISA | Negative (-) |
| 2 | HBV | Hepatitis B surface antigen (HBsAg) | ELISA | Negative (-) |
| 3 | HCV | Hepatitis C Virus antibody (HCV-IgG) | ELISA | Negative (-) |
| 4 | HTLV | Human T-cell leukemia virus antibody | ELISA | Negative (-) |
| 5 | EBV | Epstein-Barr virus capsid antigen antibody (VCA) IgM | ELISA | Negative (-) |
| 6 | CMV | Cytomegalovirus IgM antibody | ELISA | Negative (-) |
| 7 | TP | Treponema pallidum tolulized red unheated serum test (TRUST) | TPPA | Negative (-) |
